# Sphingosine-1-phosphate promotes osteogenesis by stimulating osteoblast growth and neovascularization in a vascular endothelial growth factor–dependent manner

**DOI:** 10.1093/jbmr/zjae006

**Published:** 2024-01-24

**Authors:** Annalena Wille, Sarah Weske, Karin von Wnuck Lipinski, Philipp Wollnitzke, Nathalie H Schröder, Nadine Thomas, Melissa K Nowak, Jennifer Deister-Jonas, Björn Behr, Petra Keul, Bodo Levkau

**Affiliations:** Institute of Molecular Medicine III, Heinrich Heine University Düsseldorf, 40225 Düsseldorf, Germany; Institute of Molecular Medicine III, Heinrich Heine University Düsseldorf, 40225 Düsseldorf, Germany; Institute of Molecular Medicine III, Heinrich Heine University Düsseldorf, 40225 Düsseldorf, Germany; Institute of Molecular Medicine III, Heinrich Heine University Düsseldorf, 40225 Düsseldorf, Germany; Institute of Molecular Medicine III, Heinrich Heine University Düsseldorf, 40225 Düsseldorf, Germany; Institute of Molecular Medicine III, Heinrich Heine University Düsseldorf, 40225 Düsseldorf, Germany; Institute of Molecular Medicine III, Heinrich Heine University Düsseldorf, 40225 Düsseldorf, Germany; Institute of Molecular Medicine III, Heinrich Heine University Düsseldorf, 40225 Düsseldorf, Germany; Department of Plastic Surgery, University Hospital BG Bergmannsheil, 44789 Bochum, Germany; Institute of Molecular Medicine III, Heinrich Heine University Düsseldorf, 40225 Düsseldorf, Germany; Institute of Molecular Medicine III, Heinrich Heine University Düsseldorf, 40225 Düsseldorf, Germany

**Keywords:** analysis/quantification of bone—bone μCT, animal models—genetic animal models, bone modeling and remodeling—molecular pathways-remodeling, cells of bone—osteoblasts, systems biology – bone interactors—bone-endothelial cell interaction

## Abstract

Sphingosine-1-phosphate (S1P) plays multiple roles in bone metabolism and regeneration. Here, we have identified a novel S1P-regulated osteoanabolic mechanism functionally connecting osteoblasts (OBs) to the highly specialized bone vasculature. We demonstrate that S1P/S1PR3 signaling in OBs stimulates vascular endothelial growth factor a (VEGFa) expression and secretion to promote bone growth in an autocrine and boost osteogenic H-type differentiation of bone marrow endothelial cells in a paracrine manner. VEGFa-neutralizing antibodies and VEGF receptor inhibition by axitinib abrogated OB growth in vitro and bone formation in male C57BL/6J in vivo following S1P stimulation and S1P lyase inhibition, respectively. Pharmacological S1PR3 inhibition and genetic S1PR3 deficiency suppressed VEGFa production, OB growth in vitro, and inhibited H-type angiogenesis and bone growth in male mice in vivo. Together with previous work on the osteoanabolic functions of S1PR2 and S1PR3, our data suggest that S1P-dependent bone regeneration employs several nonredundant positive feedback loops between OBs and the bone vasculature. The identification of this yet unappreciated aspect of osteoanabolic S1P signaling may have implications for regular bone homeostasis as well as diseases where the bone microvasculature is affected such as age-related osteopenia and posttraumatic bone regeneration.

## Lay Summary

Sphingosine-1-phosphate (S1P) is a signaling lipid that regulates bone growth and regeneration. In the present study, a novel regenerative mechanism was connected to S1P signaling within the bone. Activation of its receptor S1PR3 in bone-forming osteoblasts led to secretion of vascular endothelial growth factor a (VEGFa), the most potent vessel-stimulating factor. This stimulated the development of specialized vessels of the bone marrow, the H-type vessels, that supported overall bone regeneration. These findings foster our understanding of regular bone metabolism and suggest that S1P-based drugs may help treat diseases such as age-related osteopenia and posttraumatic bone regeneration, conditions crucially dependent on functional bone microvasculature.

## Introduction

Sphingosine-1-phosphate (S1P), a bioactive sphingolipid, is a well-known regulator of bone homeostasis and development. Several cells involved in bone biology have the ability to synthesize, release, and respond to S1P through its 5 receptors, S1PR1–5, ^1^ and thereby impact various cellular processes in bone homeostasis. Sphingosine-1-phosphate has also been suggested as a causal player in the pathogenesis of bone diseases. Studies in patients with osteoporosis have positively associated plasma S1P levels with the prevalence of fractures, low BMD,^2-5^ bone resorption markers,^3^ and poor responses to bisphosphonates.^6^ Others have found no association between plasma S1P carried by high- and low-density lipoproteins and hip fractures in postmenopausal women^7^ or even reduced bone marrow S1P levels in patients with osteoporotic fractures.^8^ In contrast, we have demonstrated positive correlations between serum S1P and bone formation markers and calcium, and a negative correlation with parathyroid hormone, respectively, in a large population-based study with over 4000 individuals from the SHIP-Trend cohort.^9^ Furthermore, the ratio between plasma and bone marrow S1P has been proposed as a critical factor.^10^ Therefore, systemic and local S1P effects on bone homeostasis need to be considered together with the role of individual S1P-generating and -sensing cell types in the bone microenvironment.

The effects of S1P on osteoclasts (OCs) and osteoblasts (OBs) have been studied extensively in the past. OCs have been found to predominantly express S1PR1 and S1PR2.^11^ Conditional S1PR1 knockout in OCs and monocytes prevents the exit of circulating osteoclast progenitors (OCPs) from bone to circulation. This results in enhanced local OC differentiation and hence bone resorption.^11^ In contrast, knockout of S1PR2 in OCs has been associated with reduced bone resorption^12^ and increased bone density in ovariectomy-induced osteoporosis.^13^ OBs also sense and react to S1P in numerous ways. S1P has been shown to promote OB matrix mineralization by upregulating alkaline phosphatase through PI3K/Akt signaling.^14^ The S1PR1 signaling promotes OB proliferation, whereas S1PR2 has been shown to be crucial for the recruitment of OB precursors.^13^^,^^15^ S1PR2 regulates the expression of Osterix (*Sp7*), a key transcription factor in osteoblastogenesis resulting in osteopenia in S1PR2 knockout mice.^9^ Additionally, S1PR3 signaling has been associated with calcification and matrix secretion in OBs,^16^ as confirmed by enhanced age-related osteoporosis in S1PR3 knockout mice.^17^ Furthermore, S1P has been suggested as a key player in OB-OC cross talk^18^: OC cathepsin K (*Ctsk*), a protein that plays a significant role in bone remodeling and resorption, induces the expression of sphingosine kinase 1 (*Sphk1*), the enzyme responsible for S1P generation, and the subsequently secreted S1P, in turn, promotes OB mineralization.^19^ Vice versa, S1P potently induces osteoprotegerin (*Opg*) in OBs and thereby inhibits osteoclastogenesis.^9^

Given its multiple roles in bone homeostasis, interfering with S1P signaling is a promising target for bone regeneration. We have recently shown potent osteoanabolic effects of endogenous S1P in hormone-induced osteopenia and osteoprotegerin deficiency–associated osteoporosis after raising its levels by pharmacological or genetic suppression of its degrading enzyme, S1P lyase.^9^^,^^20^ We have also identified S1PR2 as an important mediator of the autonomous osteoanabolic effects of S1P in OBs and demonstrated that a pharmacological S1PR2 agonist protects against ovariectomy-induced osteopenia.^9^^,^^20^

The effects of S1P on other cell types in bone, such as the endothelial cells (ECs) of the microvasculature, have not been investigated. This is of particular importance as there is a close functional relationship between the bone vasculature and bone homeostasis,^21^ and S1P has numerous effects on angiogenesis, vascular morphogenesis, and blood vessel development.^22-24^ Highly specialized bone vessels have been discovered and characterized in a series of elegant studies by the Ralf Adams’ laboratory. In particular, a specific vessel subtype with unique pro-osteogenic properties has been identified, the H-type vessels. They are characterized by high endomucin (*Emcn*) and CD31 (*Pecam1*) expression, have a distinct localization pattern, and associate with osteoprogenitors in the bone marrow to stimulate bone growth.^25^^,^^26^ Endothelial cells of H-type bone marrow vessels secrete numerous pro-osteogenic factors such as fibroblast growth factor 1, transforming growth factor beta 1 (*Tgfb1*), and delta-like protein 4,^27-29^ and reduced H-type vessels were observed with increasing age, leading to a diminished association with osteoprogenitors and a reduction in bone volume.^28^ Indeed, interventions that increased the number of H-type vessels in ovariectomized mice restored bone growth and counteracted postmenopausal osteoporosis.^30^^,^^31^

In this study, we hypothesized that S1P may play a functional role in the highly specialized bone vasculature and thereby affects bone regeneration. We show that S1P influences the cross talk between ECs and OBs, identify the receptors and mediators involved in this cross talk, and demonstrate how this novel interaction contributes to the osteoanabolic effects of S1P.

## Results

### Inhibition of the S1P lyase leads to an induction of pro-osteogenic H-type vessel formation in the bone marrow

**Figure 1:**
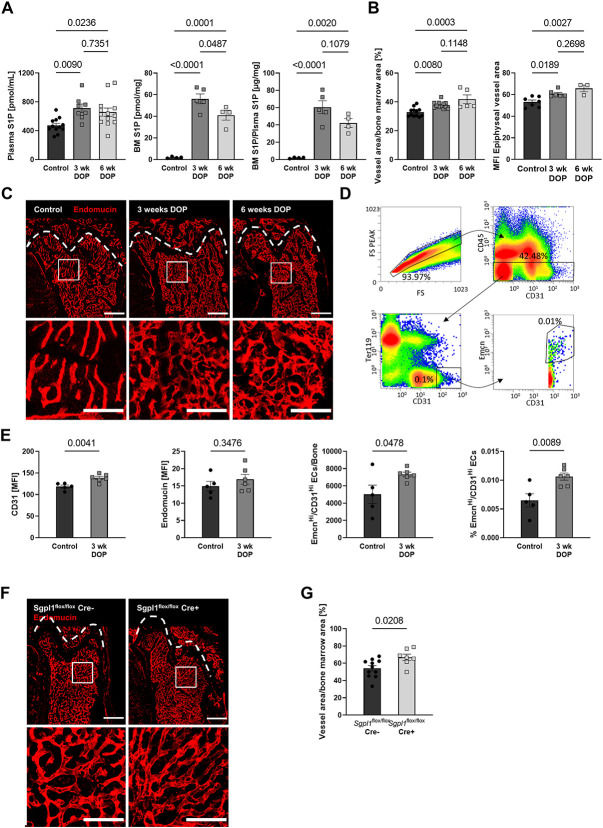
Increasing S1P levels by pharmacological inhibition of the S1P lyase or genetic deletion of the enzyme increases bone vascular density and promotes pro-osteogenic H-type endothelial cell formation. **(A)** S1P plasma levels of C57BL/6J mice after 3 and 6 weeks (wk) of DOP treatment (3 mg/kg/d) and untreated controls (*n* = 12/8/12), bone marrow (BM) S1P levels and BM/Plasma S1P ratios of these mice (*n* = 4/5/4) **(B)** quantification of vascular area per total bone marrow area in femoral bone (*n* = 12/12/6) and mean fluorescence intensity (MFI) of endomucin-stained vessels within the metaphyseal area (*n* = 7/6/3), **(C)** representative images; scale bars = 500 μm (top) and 200 μm (bottom). **(D)** Representative images and gating strategy of CD45^−^, CD31^+^, and Emcn^+^ bone marrow endothelial cells as analyzed by flow cytometry, **(E)** quantification of endothelial CD31 MFI, endomucin MFI, Emcn^HI^/CD31^HI^ H-type endothelial cells per bone, and percentage of total bone marrow cells (*n* = 5/6), **(F)** representative images of the vasculature within the femoral bone of *Sgpl^flox/flox^* Cre^−^ and *Sgpl^flox/flox^* Cre^+^ mice (scale bars = 500 μm [top] and 200 μm [bottom]) and **(G)** quantification of vascular area per bone marrow area (*n* = 11/7). Data are presented as mean ± SEM, one-way ANOVA (A and B), or 2-tailed *t*-test (E and G) were used for statistical analysis.

To determine the influence of pharmacological S1P lyase inhibition on the bone marrow microvasculature, 20-wk-old male C57BL/6J mice were treated with 4-deoxypyridoxine (DOP) for 3 and 6 wk. The treatment’s effectiveness was confirmed by 30% higher plasma S1P levels (Figure 1A). In contrast, S1P levels in the bone marrow were 50-fold higher than the controls (Figure 1A). Accordingly, the bone marrow S1P to plasma S1P ratio, which has been shown to correlate with bone mass in humans and to decrease in osteoporosis,^8^^,^^10^ was greatly increased upon DOP treatment (Figure 1A). Plasma levels of other sphingolipids such as sphingosine and sphingomyelin were increased 2–3-fold, whereas phosphatidylcholine and lysophosphatidylcholine were unchanged (Supplementary Figure S1).

Analysis of femoral bone sections revealed a 15% increase in endomucin-positive vessel area and a 14% increase in the mean fluorescence intensity (MFI) of endomucin-stained vessels in the medullary region of the bone after 3 as well as 6 wk of DOP treatment (Figure 1B and C). This was accompanied by an increase in total vessel length and branching (data not shown). These data suggested a phenotypic switch of bone marrow endothelial cells (BMECs) toward osteogenic H-type vessels.^25^^,^^32^ CD31 staining was barely detectable at this age as previously shown.^25^ To confirm the phenotypic switch, we isolated and analyzed BMECs from control and DOP-treated mice by flow cytometry. BMECs were characterized as CD45^−^, Ter119^−^, and CD31^+^ cells (Figure 1D). The MFI analysis revealed higher endomucin and CD31 expression, and an increase in total and relative numbers of Emcn^HI^/CD31^HI^ BMECs after DOP treatment (Figure 1E), thus confirming the augmented presence of H-type vessels.

As DOP inhibits the S1P lyase through its action as a vitamin B6 antagonist, we examined the bone marrow vasculature in mice conditionally lacking the S1P lyase. Indeed, *Sgpl*^flox/flox^ Cre^+^ mice exhibited 23% higher bone marrow vascular density than *Sgpl*^flox/flox^ Cre^–^ littermate controls, as assessed by endomucin-positive vessel area, 8 wk after tamoxifen-induced Cre induction (Figure 1F and G).

### Formation of H-type vessels precedes the increase in bone volume induced by S1P lyase inhibition

**Figure 2:**
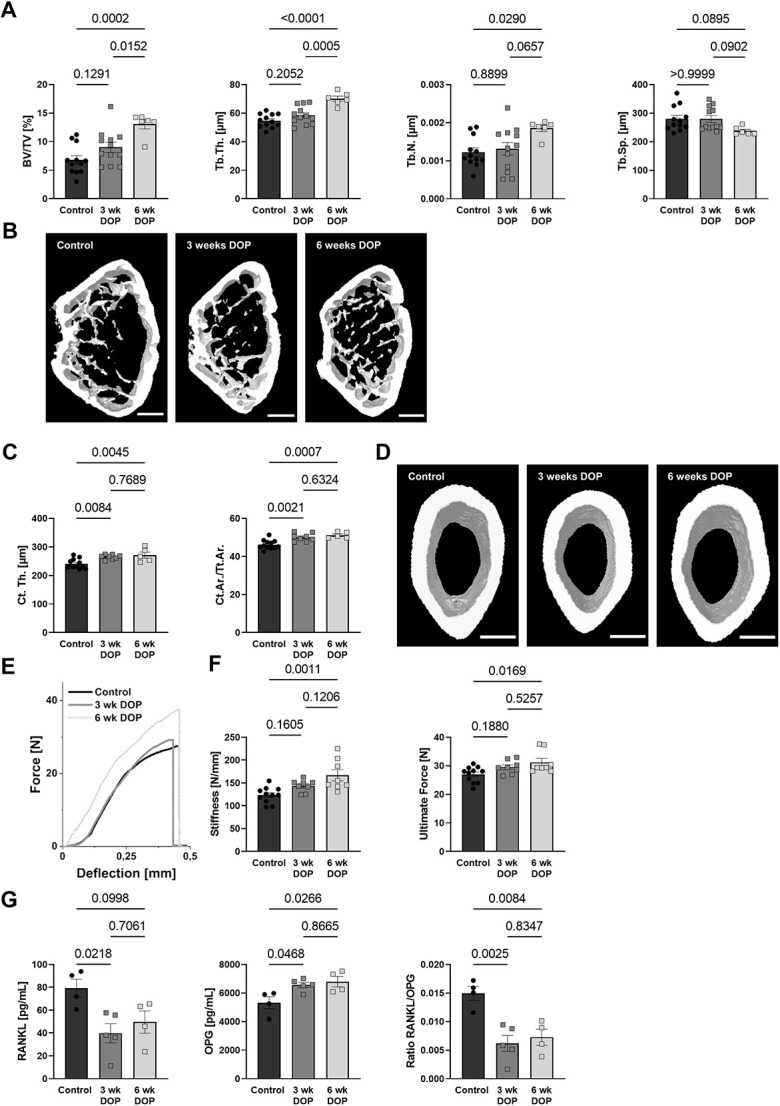
S1P-mediated changes in the bone vasculature are followed by an increase in bone mass. **(A)** BV/TV, Tb.Th., Tb.N., and Tb.Sp. measured by μCT analysis of C57BL/6J mice after 3 and 6 wk of DOP treatment (3 mg/kg/d) and untreated controls (*n* = 12/12/6) and **(B)** representative images; scale bar = 500 μm. **(C)** Quantification of cortical thickness (*n* = 12/8/5) and cortical area per total tissue area (Ct.Ar./Tt.Ar); **(D)** representative images; scale bar = 500 μm. **(E)** Representative 3-point bending test graphs and quantification of **(F)** stiffness and ultimate force (*n* = 11/8/8). **(G)** RANKL and OPG plasma levels and the resulting RANKL/OPG ratio measured using ELISA (*n* = 4/5/4). Data are presented as mean ± SEM; one-way ANOVA was used for statistical analysis.

The observation that S1P lyase inhibition promotes the generation of pro-osteogenic H-type bone microvessels raised the question of whether this might coincide with or precede the osteoanabolic effect of S1P.^9^ Microcomputed tomography (μCT) analysis of the femoral metaphyseal region of the same mice showed no increase in bone volume/total volume (BV/TV) after 3 wk and a 1.92-fold increase after 6 wk of treatment (Figure 2A and B). Similar observations held true for trabecular thickness (Tb.Th.) and trabecular number (Tb.N.) (Figure 2A and B). These data demonstrated that the S1P-mediated effects on the bone microvasculature preceded its osteogenic effects.

Changes in the cortical thickness (Ct.Th.) of these mice were already detected after 3 wk of treatment, further increasing up to 13% after 6 wk. Cortical changes are further confirmed by an increase in cortical area per total tissue area (Ct.Ar./Tt.Ar.) after 3 and 6 wk of DOP treatment (Figure 2C and D). However, this translated in higher bone strength after 6 wk but not yet 3 wk as measured by stiffness and ultimate force in 3-point bending tests (Figure 2E and F). We also measured receptor activator of NF-κB ligand (RANKL) and osteoprotegerin (OPG) plasma levels and observed a shift toward increased bone formation as evidenced by decreased RANKL, increased OPG, and a clear reduction in the RANKL/OPG ratio (Figure 2G). Similar changes in bone volume and vessel area were also detected in female mice upon DOP treatment showing a sex-independent S1P effect (Supplementary Figure S2).

### S1P induces OB calcification in a VEGFa-dependent manner, whereas OB-derived VEGFa promotes secretion of pro-osteogenic factors in BMECs

**Figure 3:**
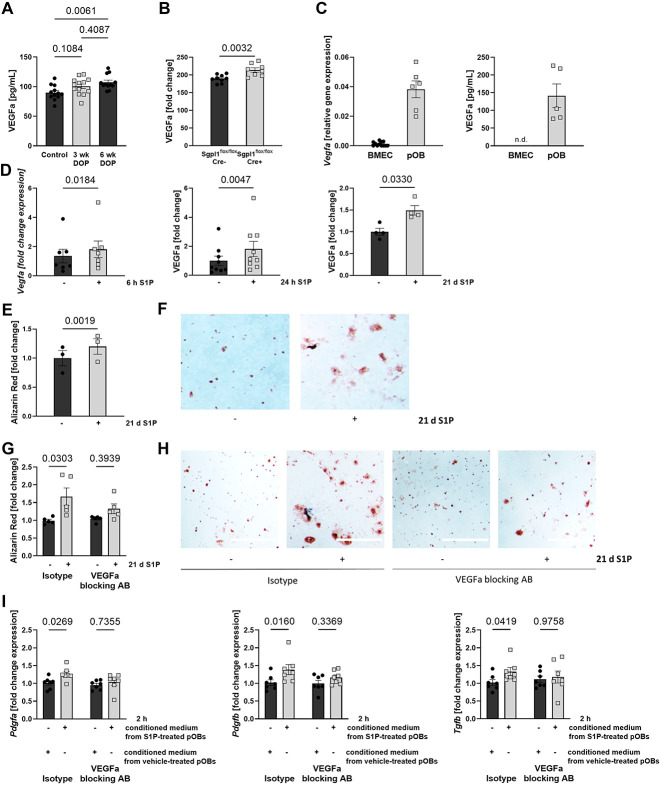
S1P stimulates calcification in osteoblasts and secretion of endothelial pro-osteogenic factors in a VEGFa-dependent manner. **(A)** VEGFa plasma concentration of male C57BL/6J mice after DOP treatment (3 mg/kg/d) for 6 wk (*n* = 11/12) and **(B)** *Sgpl^flox/flox^* Cre^−^ and *Sgpl^flox/flox^* Cre^+^ (*n* = 9/8). **(C)** Relative *Vegfa* gene expression of BMECs and pOBs (*n* = 12/6), and VEGFa protein levels in BMEC and pOB supernatants after 24 h of culture (*n* = 5/5). **(D)** Fold change gene expression of *Vegfa* in pOB after 6 h of 1 μM S1P stimulation, VEGFa levels in the supernatant of pOBs after 24 h (*n* = 9/9), and 21 d (*n* = 4/4) of 1 μM S1P stimulation. **(E)** Quantification of calcified nodules (*n* = 3/3) and **(F)** representative images after 21 d of S1P treatment; scale bar = 1 mm. **(G)** Quantification of calcified nodules formed by pOBs after 21 d of differentiation with daily 1 μM S1P or vehicle treatment in addition to 1 μg/mL VEGFa-blocking antibody or isotype control antibody (*n* = 5/5/5/5) and **(H)** representative images; scale bars = 1 mm. **(I)** Fold change expression of *Pdgfa*, *Pdgfb*, and *Tgfb1* in BMECs after 2 h of incubation in conditioned media from 1 μM S1P-treated or vehicle-treated pOBs for 7 d that was subsequently supplemented with 1 μg/mL VEGFa-blocking antibody or isotype control prior to BMECs stimulation (*n* = 7/7/7/7). Data are presented as mean ± SEM, one-way ANOVA (A), 2-tailed *t*-test (B), paired 2-tailed *t*-test (C–E), or paired 2-way ANOVA (G and I) were used for statistical analysis.

The VEGFa is a key regulator of angiogenesis and osteogenesis.^33^^,^^34^ We thus measured plasma VEGFa levels and observed them to be clearly elevated with DOP treatment compared to controls (Figure 3A), which was confirmed in *Sgpl^flox/flox^* Cre^+^ mice compared to Cre^−^ littermate controls (Figure 3B). To characterize VEGFa sources specifically in the bone marrow, we examined *Vegfa* gene expression and protein secretion in primary mouse BMECs and primary mouse osteoblasts (pOBs). We observed that *Vegfa* transcription in cells and VEGFa protein levels in supernatants were much higher in pOBs compared to BMECs after 24 h of cell culture (Figure 3C). Most importantly, S1P treatment potently induced *Vegfa* gene expression in pOBs (Figure 3D). This translated into increased VEGFa protein secretion as measured in conditioned media after 24 h and after long-term culture in the presence of S1P for 21 d (Figure 3D).

S1P is known to promote osteoblastogenesis and calcification in cultured pOBs.^14^ As we and others have previously observed, S1P potently promoted pOBs calcification as assessed by Alizarin red staining (Figure 3E and F). To investigate whether VEGFa causally contributed to this effect, we induced calcification in the presence of S1P and either a specific VEGFa-blocking antibody or its isotype control. As a result, the S1P effect was abolished by VEGFa-blockade but not with the isotype control (Figure 3G and H). These results demonstrated that S1P-mediated VEGFa production contributed to S1P’s osteogenic effect in pOBs in vitro.

Cross-talk between pOBs and BMECs is highly important and promotes each cell type’s function.^25^^,^^29^ We therefore examined the effect of pOB-derived VEGFa secreted after S1P stimulation on the phenotype of BMECs. To do this, we transferred preconditioned media from pOBs cultured with or without daily S1P supplementation for 7 d to cultured BMECs in the presence of the VEGFa-blocking antibody or its isotype control. We observed that conditioned media from S1P-treated pOBs clearly induced the expression of the pro-osteogenic factors platelet-derived growth factor A (*Pdgfa*), platelet-derived growth factor B (*Pdgfb*), and *Tgfb1* as compared to vehicle-treated controls. This induction was abolished by the VEGFa-blocking antibody but not its isotype control (Figure 3I).

The mutual effect of BMEC and pOBs was further evaluated in a coculture setting (Supplementary Figure S3). BMECs and pOBs clearly enhanced each other’s growth and formation of secondary structures such as vessel- and branchlike structures never observed in monoculture with clustering of pOBs and BMECs. The S1P facilitated tube formation and branching throughout the entire 72-h observation period with effects occurring much earlier and more extensively in its presence.

### VEGFR blockade by axitinib in vivo reveals VEGF-dependent effects of S1P lyase inhibition on bone vasculature and bone structure

**Figure 4:**
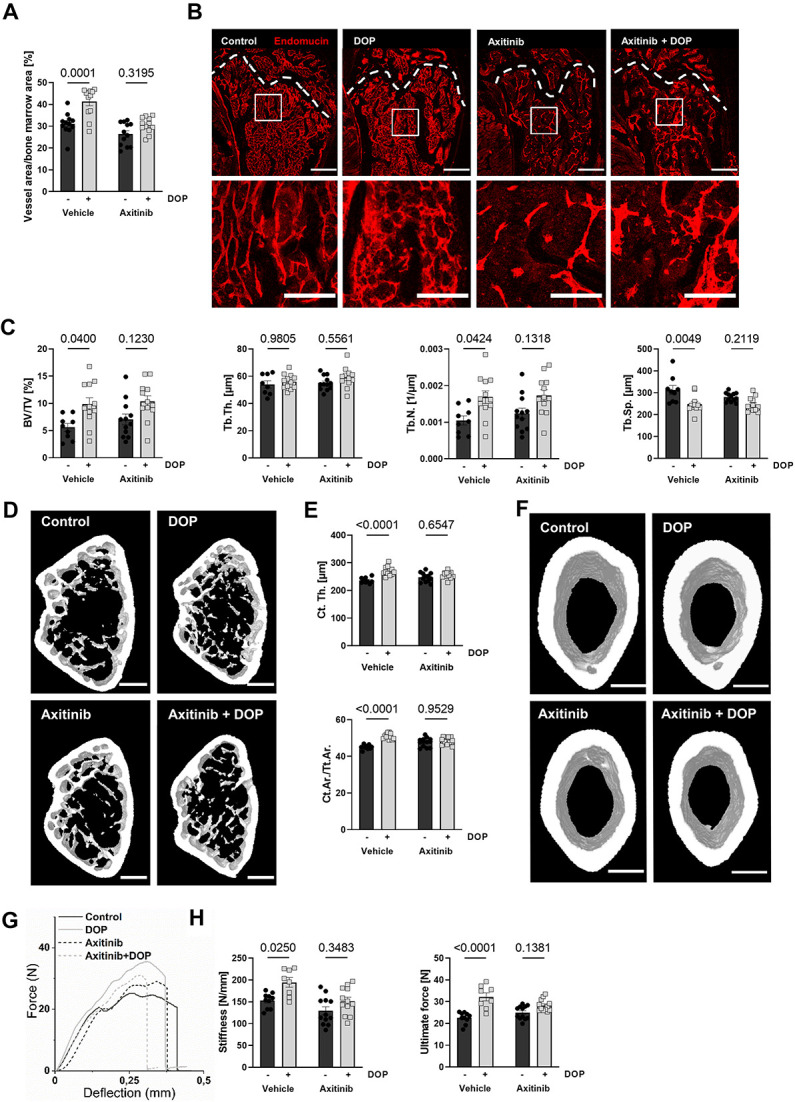
VEGF receptor inhibition through axitinib prevents the increase in vessel density and bone strengthening observed through DOP treatment. **(A)** Quantification of vascular area of endomucin-stained femoral metaphysis of male C57Bl6/J6 mice treated with DOP (3 mg/kg/d), axitinib (25 mg/kg/d), or a combination of both for 6 wk compared to untreated control animals (*n* = 12/12/12/12) and **(B)** representative images; scale bars = 500 μm (top) and 200 μm (bottom). **(C)** Quantification of bone BV/TV, Tb.Th., Tb.N., and Tb.Sp. assessed by μCT analysis of the femoral metaphysis of these mice (*n* = 9/12/12/12) and **(D)** representative μCT images; scale bar = 500 μm. **(E)** Cortical thickness of femoral bones and cortical area per total tissue area (Ct.Ar./Tt.Ar.); **(F)** representative images; scale bar = 500 μm. **(G)** Representative 3-point bending test graphs of femoral bones, **(H)** ultimate force and stiffness of femurs calculated according to 3-point bending test (*n* = 10/8/12/12). Data are presented as mean ± SEM; 2-way ANOVA was used for statistical analysis.

To elucidate the causal contribution of S1P-induced VEGF signaling on the vascular and osteoanabolic effects of S1P lyase inhibition, we treated C57BL/6J mice with DOP for 6 wk in the presence or absence of axitinib, a tyrosine kinase inhibitor with the highest inhibitory capacity against VEGFR1-3.^35^ Evaluation of thick endomucin-stained femoral bone sections demonstrated that axitinib treatment abrogated the DOP-mediated increase in vascular density compared to the vehicle-treated controls (Figure 4A and B). Furthermore, axitinib attenuated DOP-mediated increases in trabecular bone volume as assessed by BV/TV and Tb.N. and the decrease in Tb.Sp. (Figure 4C and D). These effects extended to the corticalis as well: axitinib prevented the increase in Ct.Th. and Ct.Ar./Tt.Ar. by DOP treatment (Figure 4E and F) as well as the increases in stiffness and bone strength (ultimate force) as measured by 3-point bending tests (Figure 4G and H). In summary, these results support the notion that S1P-induced VEGFa signaling contributed to the S1P-driven phenotypic switch in the bone vasculature and the changes in bone structure and biomechanical properties.

### S1P leads to OB VEGFa production and calcification in a S1PR3-dependent manner

**Figure 5:**
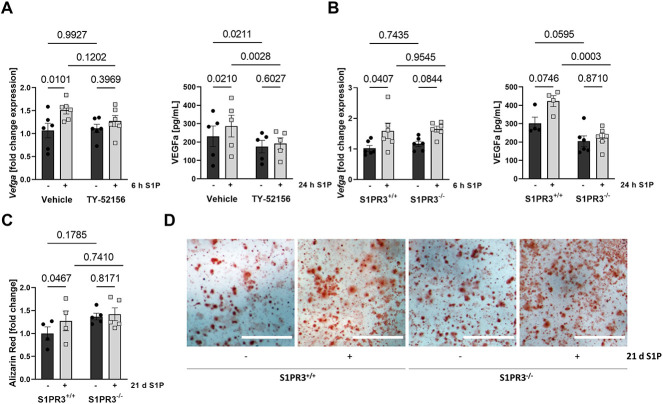
S1P-mediated VEGFa production and calcification are S1PR3 dependent in vitro. **(A)** Fold change of *Vegfa* expression in pOBs after 6-hour treatment with 1 μM S1P or vehicle control in the presence or absence of 10 μM TY-5256 (*n* = 6/6/6/6) and its respective vehicle control and VEGFa protein levels in the cell supernatant after 24 h of treatment (*n* = 5/5/5/5). **(B)** Fold change of *Vegfa* expression in S1PR3^+/+^ and S1PR3^−/−^ pOBs after 6-h treatment with 1 μM S1P or vehicle (*n* = 6/6/6/6) and VEGFa levels in the cell supernatant after 24 h of treatment (*n* = 4/4/6/6). **(C)** Quantification of calcified nodules formed by S1PR3^+/+^ and S1PR3^−/−^ pOBs after 21 d of differentiation with daily 1 μM S1P or vehicle treatment (*n* = 4/4/4/4) and **(D)** representative images; scale bars = 1 mm. Data are presented as mean ± SEM. Paired 2-way ANOVA was used for statistical analysis.

In cultured pOB, we observed that the S1PR3 antagonist TY-52156 completely abrogated *Vegfa* gene expression and protein secretion in pOB after S1P treatment (Figure 5A). Importantly, these findings were confirmed using pOBs isolated from S1PR3-deficient mice (Figure 5B). Interestingly, a decrease in VEGFa levels was observed in S1PR3-inhibited and -deficient pOBs already on a basal level when compared to control pOBs (Figure 5A and B). Furthermore, calcification experiments with long-term S1P administration (21 d) demonstrated that S1PR3^−/−^ pOB did not calcify in contrast to the robust calcification observed in S1PR3^+/+^ pOB (Figure 5C and D). The S1P did not induce RUNX2 (*Runx2*) gene expression but that of its direct transcriptional targets osteocalcin (*Bglap*) and osteopontin (*Spp1*) as well as that of osteonectin (*Sparc*) and periostin (*Postn*), and this was all absent in S1PR3^−/−^ pOBs (Supplementary Figure S4). These data demonstrated a clear dependence of S1P-mediated VEGFa production and calcification, respectively, on OB S1P/S1PR3 signaling.

### S1PR3 deficiency abrogates the effects of S1P lyase inhibition on VEGFa, H-type vessels, and osteogenesis in vivo

**Figure 6:**
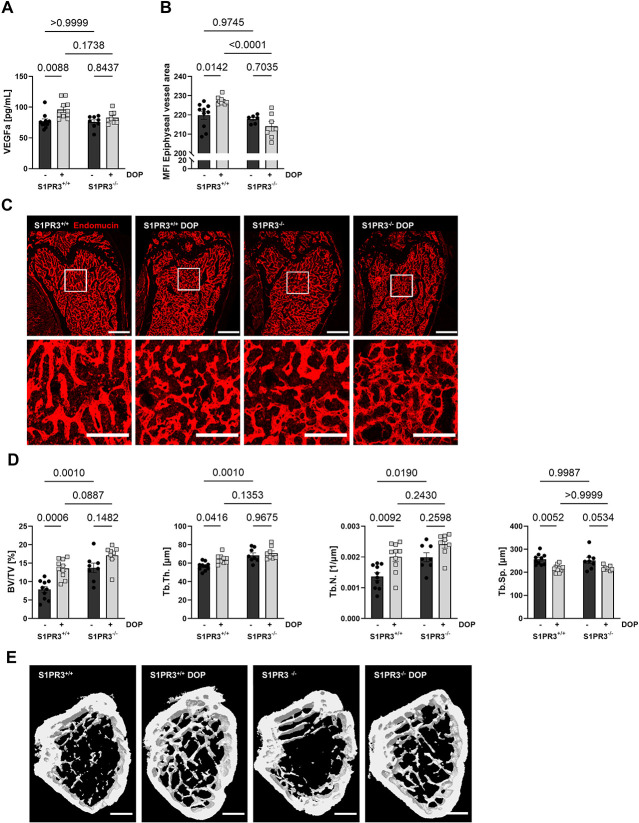
DOP-mediated VEGFa production and vascular and trabecular changes are S1PR3 dependent in vivo. **(A)** VEGFa plasma concentrations of male S1PR3^+/+^ and S1PR3^−/−^ mice treated with DOP (3 mg/kg/d) for 6 wk compared to untreated control mice (*n* = 10/10/8/8). **(B)** Quantification of MFI of endomucin-stained vessels in the femoral metaphysis (*n* = 10/9/6/7). **(C)** Representative images; scale bars = 500 μm (top) and 200 μm (bottom). **(D)** Quantification of bone BV/TV, Tb.Th., Tb.N., and Tb.Sp. as assessed by μCT analysis of the femoral metaphysis of same mice (*n* = 10/10/8/8). **(E)** Representative μCT images; scale bar = 500 μm. Data are presented as mean ± SEM. Two-way ANOVA was used for statistical analysis.

To address the role of S1PR3 in VEGFa-dependent S1P effects in vivo, we treated S1PR3^+/+^ and S1PR3^−/−^ mice with DOP for 6 wk. Although S1PR3^+/+^ mice showed the expected increase in VEGFa plasma levels with DOP treatment (1.25-fold), S1PR3^−/−^ mice did not (Figure 6A). Furthermore, endomucin staining for H-type vessels showed an increase of endomucin MFI in the bone vasculature of DOP-treated S1PR3^+/+^ but not S1PR3^−/−^ mice. This is additionally confirmed by the significant increase in endomucin staining intensity in DOP-treated wild-type mice compared to DOP-treated S1PR3^−/−^ animals (Figure 6B and C). Finally, structural bone parameters (BV/TV, Tb.Th., Tb.N.) increased with DOP treatment in S1PR3^+/+^ but not S1PR3^−/−^ mice (Figure 6D and E). Additionally, a decrease was observed in Tb.Sp. after DOP treatment, which was not significant in S1PR3^−/−^ mice (Figure 6D and E). These findings suggested that the effect of S1P-induced VEGFa signaling was initiated by S1PR3 signaling also in vivo.

Surprisingly, there was an increase in bone parameters (BV/TV, Tb.Th., and Tb.N.) in S1PR3^−/−^ compared to S1PR3^+/+^ mice (Figure 6D and E). This suggested that there may be another mechanisms by which S1PR3 actually suppresses bone mass. Indeed, osterix (*Sp7*) gene expression was clearly induced in S1PR3^−/−^ pOBs and that of osteopontin (*Spp1*) and osteonectin (*Sparc*) reduced, respectively, whereas osteocalcin (*Bglap*) and periostin (*Postn*) remained unaffected (Supplementary Figure S4). Particularly, the induction of osterix in S1PR3^−/−^ pOBs suggested enhanced differentiation. We also measured RANKL and OPG plasma levels in S1PR3^+/+^ and S1PR3^−/−^ mice and observed that the DOP-mediated RANKL decrease and OPG increase, respectively, which we had discovered before are actually S1PR3 dependent, as these effects were absent in S1PR3^−/−^ mice (Supplementary Figure S5).

## Discussion

**Figure 7:**
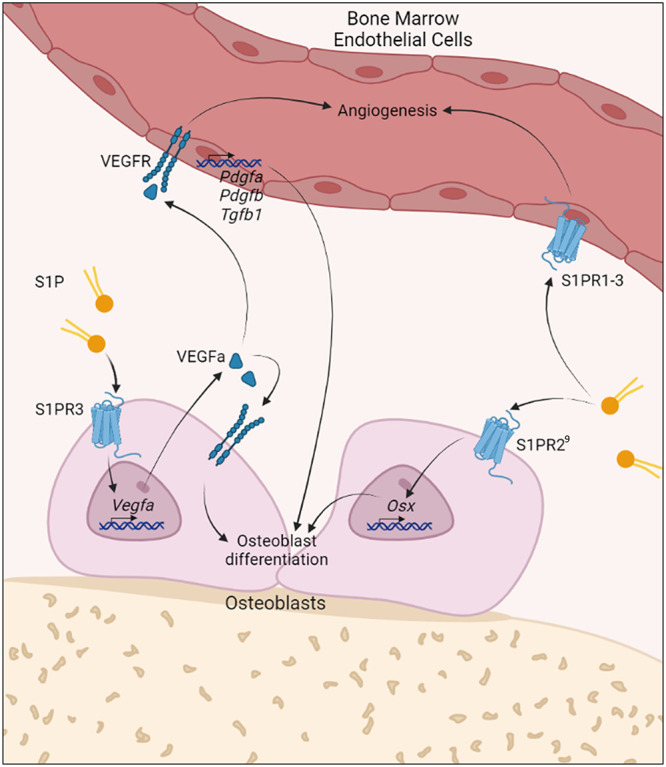
Schematic of S1P-dependent cross talk between osteoblasts and bone marrow endothelial cells acting in an autocrine and paracrine manner to stimulate bone growth. S1P stimulates VEGFa production in osteoblasts via S1PR3 to increase bone mass in an autocrine and H-type vessel formation in a paracrine manner. To this end, S1P induces VEGFa production through S1PR3 signaling in OBs that leads to autocrine stimulation of OB differentiation and calcification. In addition, secreted VEGFa stimulates angiogenesis and the generation of osteogenic H-type vessels that promote osteoblastogenesis by secreting osteogenic factors such as *Pdgfa*, *Pdgfb*, and *Tgfb1.* Furthermore, S1P directly stimulates OB differentiation through S1PR2 signaling as shown before.^9^

In this study, we have identified a novel S1P-regulated osteoanabolic pathway that functionally links the bone microvasculature with the OB compartment and serves to support and enhance each other’s pro-osteogenic functions. It consists of an autocrine loop, where S1P/S1PR3 signaling stimulates VEGFa production in OBs to promote differentiation, and a paracrine loop, where OB-derived VEGFa stimulates BMECs to form osteogenic H-type vessels (Figure 7). VEGF signaling is known as major player in bone homeostasis and regeneration: (1) VEGFa expression during development positively correlates with bone formation^36^; (2) blocking VEGF signaling reduces chondrocyte recruitment, differentiation, and trabecular bone formation^37^; (3) conditional deletion of VEGFa in OBs decreases bone mass and mineralization due by less osteoprogenitors^38^^,^^39^; (4) VEGFR1 blockade reduces bone density,^38^ and (5) VEGFR2 blockade increases bone healing.^40^

Here, we show that S1P-stimulated VEGFa production in pOBs is a potent mediator of OB differentiation and bone marrow angiogenesis and identified S1PR3 as the responsible receptor based on the following findings: (1) VEGFa production was blocked by S1PR3 antagonism in vitro and in S1P lyase–inhibited S1PR3-deficient mice in vivo; (2) S1P-mediated OB mineralization was inhibited by VEGFa neutralizing antibody in vitro; and (3) VEGFR signaling blockade by axitinib suppressed the osteoanabolic and angiogenic effects of S1P lyase inhibition in vivo. S1P has been demonstrated to enhance VEGFa gene expression in ECs and osteosarcoma cells,^41-43^ and S1PR3 to promote bone matrix formation and mineralization in OBs,^17^ respectively. In light of our findings, we propose that VEGFa is an autocrine osteoanabolic mediator of S1P/S1PR3 signaling in OBs.

Several S1PR are involved in bone homeostasis and play important roles in OB and OC biology. The S1PR1 and S1PR2 regulate OC migration and OB proliferation.^11^^,^^15^ The S1PR2 reduces bone resorption and stimulates OB differentiation.^9^^,^^12^ In contrast, the role of S1PR3 has remained rather ambiguous, as deletion led to osteopenia in aged receptor knockout animals,^17^ whereas activation induced bone matrix secretion and triggered mineralization.^16^ In the present study, we have shown that S1PR3 is directly pro-osteogenic through autocrine VEFGa signaling and indirectly by stimulating bone-specific osteogenic vessels. However, its role is more complex as S1PR3-deficient mice had higher bone mass despite their irresponsiveness to bone mass increases due to S1P lyase inhibition.

Actually, this suggests that there is a mechanism by which S1PR3 may also suppress bone mass. We have observed that osterix (*Sp7*) gene expression was induced in S1PR3 knockout OBs, and osteopontin (*Spp1*) and osteonectin (*Sparc*) reduced, respectively. Although this suggests that S1PR3 inhibits OB differentiation by suppressing osterix, osteopontin is known to stimulate OC motility^44^ and trigger OC attachment to bone, thereby increasing bone resorption,^45^ whereas osteonectin is usually expressed at sites of active bone remodeling,^46^ supporting a role of S1PR3 in OC biology.

In support, the DOP-mediated RANKL decrease and OPG increase, respectively, that we discovered in this study and previously (Weske, Nat Med 2019)^9^ were absent in S1PR3^−/−^ mice. These individual contributions can only be resolved by analysis of OB- and OC-specific S1PR3 deletions in particular and every other S1PR in general, whereas redundant and nonredundant roles of S1PRs must be tackled in double and triple knockouts.

Finally, several other cell types within the bone marrow niche and participating in bone homeostasis are sensitive to S1P: (1) S1P attracts stem cells and stromal cells from the bone marrow to the site of bone formation and induces their differentiation into bone-forming cells^47^^,^^48^; (2) S1P chemoattracts mesenchymal stem cells^49^ and induces their differentiation into OBs^50^; (3) OCPs are mobilized by S1P^11^; and (4) S1P stimulates not only chondrocyte proliferation^51^ but also cartilage degradation due to decreased aggrecan secretion.^52^ Osteocytes regulate bone homeostasis through regulation of OCs via RANKL expression.^53^^,^^54^

In our study, we show that S1P-induced OB-secreted VEGFa serves as a paracrine stimulator of pro-osteogenic H-type BMECs in vitro and H-type vessel formation in vivo. Another OB-secreted factor that induces formation of H-type vessels is slit guidance ligand 3 (*Slit3*)^55^, whereas CXC ligand 9 (*Cxcl9*) inhibits H-type vessel formation by binding to and inhibiting VEGFa signaling in ECs.^56^ We have found increased *Pdgfa*, *Pdgfb*, and *Tgfb1* expression in BMECs stimulated with OB-derived VEGFa. These growth factors are among the signature factors inducing differentiation of H-type BMECs and contribute to their capacity for osteogenic programming.^25^ Notably, OCs have also been shown to stimulate H-type vessel formation through secretion of *Pdgfb*.^57^

An interesting question is related to the source of S1P that stimulates OBs in the bone marrow. Circulating S1P may certainly be involved, but local S1P production could play a role. In addition to OBs and OCs, which are known to produce^16^ and employ S1P as an OB-OC cross talk factor,^18^ H-type ECs may themselves be a S1P source since the major physiological stimulus for generation and maintenance of H-type vessels—the high shear rate of blood flow in the long bone vasculature^58^—is known to stimulate S1P release in ECs from many origins.^59^ This would suggest the existence of another positive feedback loop in which H-type ECs secrete S1P to initiate signaling in adjacent OBs.

There is a large body of literature related to the crucial role of S1P in angiogenesis, vascular maturation, and endothelial barrier function in almost every vascular bed except that of bone, where this hasn’t been examined. In other vascular beds, a balance between S1PR1, S1PR2, and S1PR3 regulates sprouting angiogenesis, where sprouting is enhanced by S1PR3 signaling through Rho leading to tip cell formation, cytoskeletal rearrangements, and stabilization of newly formed vessels,^23^^,^^60^ and inhibited by S1PR1 and S1PR2.^24^^,^^61^^,^^62^ Once a proper vascular network and flow are established, circulating S1P activates endothelial S1PR1 that inhibits further sprouting in a negative feedback loop^63^ and triggers vascular junctions tightening and adherens junctions formation through MAPK and Gi.^61^^,^^64^ Here, we have shown that stimulation of S1PR3 by S1P promoted H-type bone vessel formation in vivo, identifying a novel role of S1PR3 in bone vasculature. In addition to the H-type endothelial phenotypic switch, we have also observed a general increase in vascular density in S1P lyase–inhibited mice. Any resulting increase in blood flow would presumably stimulate flow-dependent H-type vessel differentiation and their contribution to osteogenesis but also support osteogenesis in general by promoting the supply of oxygen, nutrients, vitamins, and minerals,^65^^,^^66^ and the recruitment of circulating osteoblastic lineage cells to sites of active bone formation.^67^ Indeed, we observed that S1P accelerated and amplified the formation of vessel- and branchlike structures in a coculture of OBs and BMEC.

In summary, we have identified VEGFa as an autocrine mediator of the osteoanabolic action of S1PR3 acting synergistically with autonomous S1PR2-mediated stimulation of OB differentiation and growth.^9^^,^^20^ This novel S1P/S1PR3/VEGFa signaling pathway also acts in a paracrine manner to stimulate bone vascularization and osteogenic H-type vessel differentiation. The resulting enhanced blood flow and shear would further support H-type vessel formation and stimulate flow-dependent S1P secretion in another positive feedback loop. Thus, osteogenesis appears to be supported by several S1P-initiated nonredundant mechanisms acting on different cell types. They dynamically and effectively support each other’s functions at multiple levels for the sake of bone regeneration. Understanding their individual functions and regulation may provide the basis for the development of new osteoanabolic therapies.

## Material and methods

### Animals

All animal experiments were conducted according to the Directive 2010/63/EU of the European Parliament and approved by the Landesamt für Natur, Umwelt und Verbraucherschutz Nordrhein-Westfalen, LANUV(reference number 81-02.04.2020.A007). Male 16- to 20-wk-old C57Bl/6J mice were obtained from Charles River Laboratories and bread and housed in the animal facility of Heinrich-Heine University Düsseldorf. Inducible *Sgpl1*^flox/flox^ actb-CreERT2 mice were provided by A. Billich (Novartis).^68^ Deletion of *Sgpl1* was performed using 40 mg/kg/d of tamoxifen injections for 5 consecutive days as described previously.^68^ To study S1P receptor involvement, S1pr3^tm1Rlp^ mice harboring an S1PR3^−/−^ genotype and corresponding S1PR3^+/+^ controls were used.^69^ The S1P lyase inhibitor DOP^70^ (Sigma-Aldrich) was administered by the drinking water. During DOP treatment, mice received a vitamin B6 reduced diet (Altromin) to accentuate the DOP effect. Axitinib (LC Laboratories) dissolved in polyethylene glycol 400, and acidified water or vehicle solution was injected intraperitoneally daily at a concentration of 25 mg/kg/d. Specific treatment concentrations and times are stated in the figure legends.

### Immunohistochemistry

To analyze the bone vasculature, femoral bones were prepared according to Kusumbe et al.^71^ About 40-μm-thick sections were obtained using a CM185 cryotome (Leica) and N35 blades (FEATHER). Sections were air dried and blocked with PBS containing 5% host serum and 0.1% Triton X (Sigma-Aldrich). Primary endomucin antibody (Santa Cruz Biotechnology; 1:100) was incubated overnight at 4 °C in antibody solution, containing 0.1% Triton X and 1% BSA (Serva) in PBS. Secondary antibody (Thermo Fisher Scientific; 1:1000) was added and incubated for 2 h at room temperature. The sections were mounted using Fluoromount-G (Thermo Fisher Scientific).

Images were acquired using a Zeiss 880 LSM confocal microscope (Carl Zeiss). For quantification, total vessel area per area of bone marrow and MFI of vascular endomucin staining within 3000 mm of the growth plate were analyzed using the FIJI image processing software.^72^

### Flow cytometry

For flow cytometry analysis of BMECs, single cell solution was obtained according to Langen et al.^29^ After lysis of red blood cells, using ACK lysis buffer for 2 min, cells were suspended in RPMI (Gibco Life Technologies) containing 10% FBS (Gibco Life Technologies). Cells were blocked in PBS containing 1% BSA, 0.01% sodium azide (Sigma-Aldrich), and FC-Block (BD; 1:100) for 5 min. For fluorescent labeling, CD31-AF488 (BioLegend; 1:40), Emcn-AF647 (Santa Cruz Biotechnology; 1:50), Ter119-PE (Miltenyi, Bergisch Gladbach, Germany; 1:50), and CD45.2-V500 (BD; 1:125) were added for 45 min. After thorough washing, cells were suspended in PBS containing 1% BSA and 0.01% sodium azide and acquired on the Gallios 10/3 flow cytometer (Beckmann Coulter).

### Microcomputer tomography

A SkyScan X-ray Microtomography 1072 (SkyScan, Belgium) was used to analyze femoral trabecular bone volume and Ct.Th. Images were acquired at 70 kV and 114 μA using a 180° circular acquisition with 0.45° steps between projections. Trabecular bone volume was measured at 11.32 μm pixel size and Ct.Th. at 18.88 μm pixel size.

Images were reconstructed using NRecon software (version 1.6.9.4; SkyScan). Density calibration for each sample was performed using hydroxyapatite calibration standards (phantoms) corresponding to densities of 250 and 750 mg/cm^3^, allowing for the calibration and adjustment of grayscale values. This allows the application of a threshold of 802 mg/cm^3^ for the analysis of bone. Cortical bone was analyzed in a region of 50 consecutive slices at the midpoint of the femoral bone, corresponding to a region of 0.955 mm. Trabecular bone was analyzed in a region starting 0.566 mm below the growth plate on 126 consecutive CT slices, corresponding to a region of 1.427 mm. Analysis was carried out using the CT Analyzer software (version 1.18.9.0+; SkyScan).

Studies were performed following the guidelines for the assessment of bone microstructure in rodents by means of μCT.^73^

### Three-point bending test

The Material Testing System Shimadzu EZ Test EZ-SX machine (Shimadzu) was used for mechanical testing of bones. Femora were placed on 2 support points 5 mm apart. A loading point was placed in the middle of the diaphysis. A constant load of 3 mm/min was applied using a 500 N load cell until failure occurred at the loading point. TrapeziumX software (Shimadzu) was used to measure load and displacement every 5 ms. The ultimate load (strength) is calculated from the load–displacement curve as the point at which failure occurs, and the stiffness is calculated from the slope of the curve.

### Cell culture

Primary mouse BMECs isolated from C57BL/6 mice were obtained from Cell Biologics. Primary OBs were isolated according to Declercq et al.^74^ Shortly, the hind limb tibiae and femurs of 9-d-old mice were isolated and thoroughly cleaned, epiphyseal regions and joints removed, and the diaphysis of the femurs and tibiae rinsed with prewarmed HBSS (Gibco Life Technologies) to remove bone marrow. To promote OB proliferation from mesenchymal cells and get rid of any CD31^+^ and CD45^+^ cells, bone pieces were placed on a 6-cm Petri dish and carefully covered with OB proliferation medium (DMEM, 10% FBS, 1% Antibiotic-Antimycotic Solution, 100 μM 2-phospho-l-ascorbic acid). Once the cells had reached confluence, they were used for further experiments. Isolating and comparing BMEC and primary OB from the same mouse proved unsuccessful. Thus, our compromise to use primary BMECs isolated from C57Bl6/J mice (purchased) with OBs from C57Bl6/J mice (isolated). Treatment of pOBs was performed in OB differentiation medium (α-MEM, 10% FBS, 1% Antibiotic-Antimycotic Solution, 4 mM l-glutamine, 100 μM 2-phospho-l-ascorbic acid, 10 mM β-glycerophosphate). The S1P (Enzo Life Sciences) was used for treatment at a concentration of 1 μM. Treatment with the S1P receptor 3 antagonist TY-52156 (Cayman Chemical) was performed 30 min before S1P stimulation at 10 μM. To exclude solvent effects, the appropriate solvent controls were added to the control groups. For preconditioned medium treatment, pOBs were cultured in T75 cell culture flasks and stimulated daily with 1 μM S1P or solvent control in OB differentiation medium for 7 d. Cell supernatants were then added to BMECs for indicated times. Simultaneously, purified anti-mouse VEGFa antibody or rat IgG2a isotype control (BioLegend) was used at 1 μg/mL.

### Quantitative real-time PCR

Total RNA was isolated with the innuPREP RNA Mini Kit 2.0 (Analytic Jena) following the manufacturer's protocol. cDNA synthesis of 200 ng RNA was performed using the RevertAid First Strand cDNA Synthesis Kit (Thermo Fisher Scientific). The iQ Syber Green Supermix (Bio-Rad) was used for the quantitative PCR analysis. The reaction was run for 40 cycles of 95 °C for 10 s, 55° C for 10 s, and 72 °C for 30 s. *Gapdh* is used as a reference housekeeping gene. The used primers are listed in Table 1 and Supplementary Table 1.

**Table 1:** Primers used for quantitative real-time PCR.

**Gene**	**Product/5′-3′ sequence**	**Company**
*Gapdh*	Fw: AGGTCGGTGTGAACGGATTTG	Eurofins Genomics, Ebersberg, Germany
	Rv: TGTAGACCATGTAGTTGAGGTCA	
*Pdgfa*	Fw: GAGGAAGCCGAGATACCCC	Eurofins Genomics, Ebersberg, Germany
	Rv: TGCTGTGGATCTGACTTCGAG	
*Pdgfb*	Fw: CATCCGCTCCTTTGATGATCTT	Eurofins Genomics, Ebersberg, Germany
	Rv: GTGCTCGGGTCATGTTCAAGT	
*Vegfa*	Mm_Vegfa_1_SG QuantiTect Primer Assay	Qiagen, Hilden, Germany
*Tgfb1*	Mm_Tgfb1_1_SG QuantiTect Primer Assay	Qiagen, Hilden, Germany

### VEGFa, RANKL, and OPG ELISA

VEGFa levels in plasma and cell culture supernatants were measured using the Mouse VEGF Quantikine ELISA Kit (R&D Systems). The RANKL levels were measured using the Mouse TRANCE/RANKL Quantikine Kit (R&D Systems), and OPG levels were measured using the Ancillary Kit 2 and the Duo Set Mouse Osteoprotegerin/TNFRST11b kit (R&D Systems). Blood plasma was collected using whole blood with the addition of EDTA as an anticoagulant. Blood was centrifuged at 1500× *g* for 10 min at 4 °C, and plasma was collected.

### Alizarin red staining

For Alizarin red staining, pOBs were cultured for 21 d in differentiation medium and spiked daily with 1 μM S1P, the appropriate solvent is added to control cells. For VEGFa blockade, Ultra-LEAF-purified anti-mouse VEGFa antibody (BioLegend) was used at a concentration of 1 μg/mL. Rat IgG2a, κ was used as the corresponding isotype control. The cells were fixed and stained with 1 mg/mL Alizarin red (Sigma-Aldrich) for 20 min. Alizarin red is extracted in 100 mM cetylpyridinium chloride (Sigma-Aldrich), and absorbance was measured at 570 nm.

### LC-MS/MS measurement

Plasma S1P was detected by positive electrospray ionization using an LCMS-8050 triple quadrupole mass spectrometer (Shimadzu). The following settings were used: nebulizer: 3 L/min, interface temperature: 300 °C, desolvation temperature: 526 °C, heat block temperature: 400 °C, and drying gas flow: 10 L/min. Gradient separation was performed at 40 °C using a Nexera X3 UHPLC system (Shimadzu) and a 60 × 2.0 mm MultoHigh 100 RP18 column (CS-Chromatographie Service). The gradients of the mobile phase are listed in Table 2 and are used at a flow rate of 0.4 mL/min. Data were analyzed using LabSolutions 5.114 (Shimadzu).

**Table 2:** LC-MS gradient settings.

**Time (min)**	**MeOH dilution (%)**	**1 % aq. Formic acid dilution (%)**	**Curve**
0	10	90	−2
*3*	100	0	0
*12*	100	0	0
*12.01*	10	0	0

### Statistical analysis

All data points are presented as the mean ± SD and were analyzed for statistical significance using GraphPad Prism (version 9.3.1; GraphPad). For *P* ≥ .05, groups were considered significant. Statistical tests used are indicated in the figure legends. Equal variance and normality tests were performed on all data points. For data analyzed using 2-way ANOVA, detailed statistical analysis of interaction, row, and column *P*-values can be found in Supplementary Tables S2–S5.

## Supplementary Material

wille_et_al_supplementary_information_revison3_zjae006

## Data Availability

The data that support the findings of this study are available from the corresponding author upon reasonable request.

## References

[1] Hla T. Sphingosine 1-phosphate receptors. Prostaglandins Other Lipid Media*t*. 2001;64(1-4):135–142. 10.1016/S0090-6980(01)00109-5. 11324703

[2] Lee SH, Lee SY, Lee YS, et al. Higher circulating sphingosine 1-phosphate levels are associated with lower bone mineral density and higher bone resorption marker in humans. J Clin Endocrinol Meta*b*. 2012;97(8):E1421–E1428. 10.1210/jc.2012-1044. 22679064

[3] Kim BJ, Koh JM, Lee SY, et al. Plasma sphingosine 1-phosphate levels and the risk of vertebral fracture in postmenopausal women. J Clin Endocrinol Meta*b*. 2012;97(10):3807–3814. 10.1210/jc.2012-2346. 22879631

[4] Lee SH, Lee JY, Lim KH, et al. Associations of circulating levels of sphingosine 1-phosphate with the trabecular bone score and bone mineral density in postmenopausal women. J Clin Densito*m*. 2021;24(3):414–421. 10.1016/j.jocd.2021.03.005. 33846060

[5] Ardawi MM, Rouzi AA, Al-Senani NS, Qari MH, Elsamanoudy AZ, Mousa SA. High plasma sphingosine 1-phosphate levels predict osteoporotic fractures in postmenopausal women: the Center of Excellence for Osteoporosis Research Study. J Bone Meta*b*. 2018;25(2):87–98. 10.11005/jbm.2018.25.2.87. 29900158 PMC5995758

[6] Bae SJ, Lee SH, Ahn SH, Kim HM, Kim BJ, Koh JM. The circulating sphingosine-1-phosphate level predicts incident fracture in postmenopausal women: a 3.5-year follow-up observation study. Osteoporos In*t*. 2016;27(8):2533–2541. 10.1007/s00198-016-3565-z. 26984570

[7] Song HE, Lee SH, Kim SJ, Kim BJ, Yoo HJ, Koh JM. Association of circulating levels of total and protein-bound sphingosine 1-phosphate with osteoporotic fracture. J Investig Me*d*. 2020;68(7):1295–1299. 10.1136/jim-2020-001322. 32675084

[8] Ahn SH, Koh JM, Gong EJ, et al. Association of bone marrow sphingosine 1-phosphate levels with osteoporotic hip fractures. J Bone Meta*b*. 2013;20(2):61–65. 10.11005/jbm.2013.20.2.61. 24524059 PMC3910311

[9] Weske S, Vaidya M, Reese A, et al. Targeting sphingosine-1-phosphate lyase as an anabolic therapy for bone loss. Nat Me*d*. 2018;24(5):667–678. 10.1038/s41591-018-0005-y. 29662200

[10] Kim BJ, Shin KO, Kim H, et al. The effect of sphingosine-1-phosphate on bone metabolism in humans depends on its plasma/bone marrow gradient. J Endocrinol Investi*g*. 2016;39(3):297–303. 10.1007/s40618-015-0364-x. 26219613

[11] Ishii M, Egen JG, Klauschen F, et al. Sphingosine-1-phosphate mobilizes osteoclast precursors and regulates bone homeostasis. Natur*e*. 2009;458(7237):524–528. 10.1038/nature07713. 19204730 PMC2785034

[12] Ishii M, Kikuta J, Shimazu Y, Meier-Schellersheim M, Germain RN. Chemorepulsion by blood S1P regulates osteoclast precursor mobilization and bone remodeling in vivo. J Exp Me*d*. 2010;207(13):2793–2798. 10.1084/jem.20101474. 21135136 PMC3005230

[13] Kikuta J, Kawamura S, Okiji F, et al. Sphingosine-1-phosphate-mediated osteoclast precursor monocyte migration is a critical point of control in antibone-resorptive action of active vitamin D. Proc Natl Acad Sci U S *A*. 2013;110(17):7009–7013. 10.1073/pnas.1218799110. 23569273 PMC3637769

[14] Matsuzaki E, Hiratsuka S, Hamachi T, et al. Sphingosine-1-phosphate promotes the nuclear translocation of β-catenin and thereby induces osteoprotegerin gene expression in osteoblast-like cell lines. Bon*e*. 2013;55(2):315–324. 10.1016/j.bone.2013.04.008. 23612487

[15] Tantikanlayaporn D, Tourkova IL, Larrouture Q, et al. Sphingosine-1-phosphate modulates the effect of estrogen in human osteoblasts. JBMR Plu*s*. 2018;2(4):217–226. 10.1002/jbm4.10037. 30123862 PMC6095197

[16] Brizuela L, Martin C, Jeannot P, et al. Osteoblast-derived sphingosine 1-phosphate to induce proliferation and confer resistance to therapeutics to bone metastasis-derived prostate cancer cells. Mol Onco*l*. 2014;8(7):1181–1195. 10.1016/j.molonc.2014.04.001. 24768038 PMC5528572

[17] Keller J, Catala-Lehnen P, Huebner AK, et al. Calcitonin controls bone formation by inhibiting the release of sphingosine 1-phosphate from osteoclasts. Nat Commu*n*. 2014;5(1):5215. 10.1038/ncomms6215. 25333900 PMC4205484

[18] Ryu J, Kim HJ, Chang EJ, Huang H, Banno Y, Kim HH. Sphingosine 1-phosphate as a regulator of osteoclast differentiation and osteoclast-osteoblast coupling. EMBO *J*. 2006;25(24):5840–5851. 10.1038/sj.emboj.7601430. 17124500 PMC1698879

[19] Lotinun S, Kiviranta R, Matsubara T, et al. Osteoclast-specific cathepsin K deletion stimulates S1P-dependent bone formation. J Clin Inves*t*. 2013;123(2):666–681. 10.1172/JCI64840. 23321671 PMC3561821

[20] Weske S, Vaidya M, von Wnuck Lipinski K, et al. Agonist-induced activation of the S1P receptor 2 constitutes a novel osteoanabolic therapy for the treatment of osteoporosis in mice. Bon*e*. 2019;125:1–7. 10.1016/j.bone.2019.04.015. 31028959

[21] Trueta J, Morgan JD. The vascular contribution to osteogenesis. I. Studies by the injection method. J Bone Joint Surg B*r*. 1960;42-B(1):97–109. 10.1302/0301-620X.42B1.97. 13855127

[22] Hla T, Maciag T. An abundant transcript induced in differentiating human endothelial cells encodes a polypeptide with structural similarities to G-protein-coupled receptors. J Biol Che*m*. 1990;265(16):9308–9313. 10.1016/S0021-9258(19)38849-0. 2160972

[23] Garcia JG, Liu F, Verin AD, et al. Sphingosine 1-phosphate promotes endothelial cell barrier integrity by Edg-dependent cytoskeletal rearrangement. J Clin Inves*t*. 2001;108(5):689–701. 10.1172/JCI12450. 11544274 PMC209379

[24] Del Galdo S, Vettel C, Heringdorf DM, Wieland T. The activation of RhoC in vascular endothelial cells is required for the S1P receptor type 2-induced inhibition of angiogenesis. Cell Signa*l*. 2013;25(12):2478–2484. 10.1016/j.cellsig.2013.08.017. 23993968

[25] Kusumbe AP, Ramasamy SK, Adams RH. Coupling of angiogenesis and osteogenesis by a specific vessel subtype in bone. Natur*e*. 2014;507(7492):323–328. 10.1038/nature13145. 24646994 PMC4943525

[26] Ramasamy SK, Kusumbe AP, Wang L, Adams RH. Endothelial Notch activity promotes angiogenesis and osteogenesis in bone. Natur*e*. 2014;507(7492):376–380. 10.1038/nature13146. 24647000 PMC4943529

[27] Xu C, Dinh VV, Kruse K, et al. Induction of osteogenesis by bone-targeted Notch activation. Elif*e*. 2022;11(11):e60183. 10.7554/eLife.60183. PMC8880996 35119364

[28] Kusumbe AP, Adams RH. Osteoclast progenitors promote bone vascularization and osteogenesis. Nat Me*d*. 2014;20(11):1238–1240. 10.1038/nm.3747. 25375923

[29] Langen UH, Pitulescu ME, Kim JM, et al. Cell-matrix signals specify bone endothelial cells during developmental osteogenesis. Nat Cell Bio*l*. 2017;19(3):189–201. 10.1038/ncb3476. 28218908 PMC5580829

[30] Wang L, Zhou F, Zhang P, et al. Human type H vessels are a sensitive biomarker of bone mass. Cell Death Di*s*. 2017;8(5):e2760. 10.1038/cddis.2017.36. 28471445 PMC5520742

[31] Zhang J, Pan J, Jing W. Motivating role of type H vessels in bone regeneration. Cell Proli*f*. 2020;53(9):e12874. 10.1111/cpr.12874. 33448495 PMC7507571

[32] Ramasamy SK, Kusumbe AP, Itkin T, Gur-Cohen S, Lapidot T, Adams RH. Regulation of hematopoiesis and osteogenesis by blood vessel-derived signals. Annu Rev Cell Dev Bio*l*. 2016;32(1):649–675. 10.1146/annurev-cellbio-111315-124936. 27576121

[33] Carmeliet P, Ferreira V, Breier G, et al. Abnormal blood vessel development and lethality in embryos lacking a single VEGF allele. Natur*e*. 1996;380(6573):435–439. 10.1038/380435a0. 8602241

[34] Zelzer E, McLean W, Ng YS, et al. Skeletal defects in VEGF(120/120) mice reveal multiple roles for VEGF in skeletogenesis. Developmen*t*. 2002;129(8):1893–1904. 10.1242/dev.129.8.1893. 11934855

[35] Lu L, Saha D, Martuza RL, Rabkin SD, Wakimoto H. Single agent efficacy of the VEGFR kinase inhibitor axitinib in preclinical models of glioblastoma. J Neuro-Onco*l*. 2015;121(1):91–100. 10.1007/s11060-014-1612-1. PMC4751887 25213669

[36] Rabie AB, Shum L, Chayanupatkul A. VEGF and bone formation in the glenoid fossa during forward mandibular positioning. Am J Orthod Dentofac Ortho*p*. 2002;122(2):202–209. 10.1067/mod.2002.125991. 12165776

[37] Gerber HP, Vu TH, Ryan AM, Kowalski J, Werb Z, Ferrara N. VEGF couples hypertrophic cartilage remodeling, ossification and angiogenesis during endochondral bone formation. Nat Me*d*. 1999;5(6):623–628. 10.1038/9467. 10371499

[38] Hu K, Olsen BR. Osteoblast-derived VEGF regulates osteoblast differentiation and bone formation during bone repair. J Clin Inves*t*. 2016;126(2):509–526. 10.1172/JCI82585. 26731472 PMC4731163

[39] Liu Y, Berendsen AD, Jia S, et al. Intracellular VEGF regulates the balance between osteoblast and adipocyte differentiation. J Clin Inves*t*. 2012;122(9):3101–3113. 10.1172/JCI61209. 22886301 PMC3428080

[40] Jacobsen KA, Al-Aql ZS, Wan C, et al. Bone formation during distraction osteogenesis is dependent on both VEGFR1 and VEGFR2 signaling. J Bone Miner Re*s*. 2008;23(5):596–609. 10.1359/jbmr.080103. 18433297 PMC2674537

[41] Heo K, Park KA, Kim YH, et al. Sphingosine 1-phosphate induces vascular endothelial growth factor expression in endothelial cells. BMB Re*p*. 2009;42(10):685–690. 10.5483/BMBRep.2009.42.10.685. 19874715

[42] Wang H, Cai KY, Li W, Huang H. Sphingosine-1-phosphate induces the migration and angiogenesis of Epcs through the Akt signaling pathway via sphingosine-1-phosphate receptor 3/platelet-derived growth factor receptor-β. Cell Mol Biol Let*t*. 2015;20(4):597–611. 10.1515/cmble-2015-0035. 26208383

[43] Huang CC, Tseng TT, Liu SC, et al. S1P increases VEGF production in osteoblasts and facilitates endothelial progenitor cell angiogenesis by inhibiting miR-16-5p expression via the c-Src/FAK signaling pathway in rheumatoid arthritis. Cel*l*. 2021;10(8):2168. 10.3390/cells10082168. PMC8393529 34440937

[44] Chellaiah MA, Hruska KA. The integrin alpha(v)beta(3) and CD44 regulate the actions of osteopontin on osteoclast motility. Calcif Tissue In*t*. 2003;72(3):197–205. 10.1007/s00223-002-1025-6. 12469249

[45] Sodek J, Chen J, Nagata TK, Shohei Todescan JR, Reynaldo LIWS, Kim RH. Regulation of osteopontin expression in osteoblasts. Ann N Y Acad Sc*i*. 1995;760(1):223–241. 10.1111/j.1749-6632.1995.tb44633.x. 7785896

[46] Delany AM, Amling M, Priemel M, Howe C, Baron R, Canalis E. Osteopenia and decreased bone formation in osteonectin-deficient mice. J Clin Inves*t*. 2000;105(7):915–923. 10.1172/JCI7039. 10749571 PMC377474

[47] Liu J, Hsu A, Lee JF, Cramer DE, Lee MJ. To stay or to leave: stem cells and progenitor cells navigating the S1P gradient. World J Biol Che*m*. 2011;2(1):1–13. 10.4331/wjbc.v2.i1.1. 21472036 PMC3070303

[48] Meriane M, Duhamel S, Lejeune L, Galipeau J, Annabi B. Cooperation of matrix metalloproteinases with the RhoA/Rho kinase and mitogen-activated protein kinase kinase-1/extracellular signal-regulated kinase signaling pathways is required for the sphingosine-1-phosphate-induced mobilization of marrow-derived stromal cells. Stem Cell*s*. 2006;24(11):2557–2565. 10.1634/stemcells.2006-0209. 16931773

[49] Quint P, Ruan M, Pederson L, et al. Sphingosine 1-phosphate (S1P) receptors 1 and 2 coordinately induce mesenchymal cell migration through S1P activation of complementary kinase pathways. J Biol Che*m*. 2013;288(8):5398–5406. 10.1074/jbc.M112.413583. 23300082 PMC3581421

[50] Hashimoto Y, Matsuzaki E, Higashi K, et al. Sphingosine-1-phosphate inhibits differentiation of C3H10T1/2 cells into adipocyte. Mol Cell Bioche*m*. 2015;401(1-2):39–47. 10.1007/s11010-014-2290-1. 25445169

[51] Stradner MH, Hermann J, Angerer H, et al. Sphingosine-1-phosphate stimulates proliferation and counteracts interleukin-1 induced nitric oxide formation in articular chondrocytes. Osteoarthr Carti*l*. 2008;16(3):305–311. 10.1016/j.joca.2007.06.018. 17703957

[52] Masuko K, Murata M, Nakamura H, Yudoh K, Nishioka K, Kato T. Sphingosine-1-phosphate attenuates proteoglycan aggrecan expression via production of prostaglandin E2 from human articular chondrocytes. BMC Musculoskelet Disor*d*. 2007;8(1):29. 10.1186/1471-2474-8-29. 17374154 PMC1847513

[53] Xiong J, Piemontese M, Onal M, et al. Osteocytes, not osteoblasts or lining cells, are the main source of the RANKL required for osteoclast formation in remodeling bone. PLoS On*e*. 2015;10(9):e0138189. 10.1371/journal.pone.0138189. 26393791 PMC4578942

[54] Nakashima T, Hayashi M, Fukunaga T, et al. Evidence for osteocyte regulation of bone homeostasis through RANKL expression. Nat Me*d*. 2011;17(10):1231–1234. 10.1038/nm.2452. 21909105

[55] Xu R, Yallowitz A, Qin A, et al. Targeting skeletal endothelium to ameliorate bone loss. Nat Me*d*. 2018;24(6):823–833. 10.1038/s41591-018-0020-z. 29785024 PMC5992080

[56] Huang B, Wang W, Li Q, et al. Osteoblasts secrete Cxcl9 to regulate angiogenesis in bone. Nat Commu*n*. 2016;7:13885. 10.1038/ncomms13885. 27966526 PMC5171795

[57] Xie H, Cui Z, Wang L, et al. PDGF-BB secreted by preosteoclasts induces angiogenesis during coupling with osteogenesis. Nat Me*d*. 2014;20(11):1270–1278. 10.1038/nm.3668. 25282358 PMC4224644

[58] Ramasamy SK, Kusumbe AP, Schiller M, et al. Blood flow controls bone vascular function and osteogenesis. Nat Commu*n*. 2016;7(1):13601. 10.1038/ncomms13601. 27922003 PMC5150650

[59] Venkataraman K, Lee Y-M, Michaud J, et al. Vascular endothelium as a contributor of plasma sphingosine 1-phosphate. Circ Re*s*. 2008;102(6):669–676. 10.1161/CIRCRESAHA.107.165845. 18258856 PMC2659392

[60] Wang H, Huang H, Ding SF. Sphingosine-1-phosphate promotes the proliferation and attenuates apoptosis of endothelial progenitor cells via S1PR1/S1PR3/PI3K/Akt pathway. Cell Biol In*t*. 2018;42(11):1492–1502. 10.1002/cbin.10991. 29790626

[61] Gaengel K, Niaudet C, Hagikura K, et al. The sphingosine-1-phosphate receptor S1PR1 restricts sprouting angiogenesis by regulating the interplay between VE-cadherin and VEGFR2. Dev Cel*l*. 2012;23(3):587–599. 10.1016/j.devcel.2012.08.005. 22975327

[62] Jung B, Obinata H, Galvani S, et al. Flow-regulated endothelial S1P receptor-1 signaling sustains vascular development. Dev Cel*l*. 2012;23(3):600–610. 10.1016/j.devcel.2012.07.015. 22975328 PMC3443394

[63] Ben Shoham A, Malkinson G, Krief S, et al. S1P1 inhibits sprouting angiogenesis during vascular development. Developmen*t*. 2012;139(20):3859–3869. 10.1242/dev.078550. 22951644

[64] Lee M-J, Thangada S, Claffey KP, et al. Vascular endothelial cell adherens junction assembly and morphogenesis induced by sphingosine-1-phosphate. Cel*l*. 1999;99(3):301–312. 10.1016/S0092-8674(00)81661-X. 10555146

[65] Marenzana M, Arnett TR. The key role of the blood supply to bone. Bone Re*s*. 2013;1(3):203–215. 10.4248/BR201303001. 26273504 PMC4472103

[66] Pugsley MK, Tabrizchi R. The vascular system: an overview of structure and function. J Pharmacol Toxicol Method*s*. 2000;44(2):333–340. 10.1016/S1056-8719(00)00125-8. 11325577

[67] Eghbali-Fatourechi GZ, Lamsam J, Fraser D, Nagel D, Riggs BL, Khosla S. Circulating osteoblast-lineage cells in humans. N Engl J Me*d*. 2005;352(19):1959–1966. 10.1056/NEJMoa044264. 15888696

[68] Billich A, Baumruker T, Beerli C, et al. Partial deficiency of sphingosine-1-phosphate lyase confers protection in experimental autoimmune encephalomyelitis. PLoS On*e*. 2013;8(3):e59630. 10.1371/journal.pone.0059630. 23544080 PMC3609791

[69] Kono M, Mi Y, Liu Y, et al. The sphingosine-1-phosphate receptors S1P1, S1P2, and S1P3 function coordinately during embryonic angiogenesis. J Biol Che*m*. 2004;279(28):29367–29373. 10.1074/jbc.M403937200. 15138255

[70] Schwab SR, Pereira JP, Matloubian M, Xu Y, Huang Y, Cyster JG. Lymphocyte sequestration through S1P lyase inhibition and disruption of S1P gradients. Scienc*e*. 2005;309(5741):1735–1739. 10.1126/science.1113640. 16151014

[71] Kusumbe AP, Ramasamy SK, Starsichova A, Adams RH. Sample preparation for high-resolution 3D confocal imaging of mouse skeletal tissue. Nat Proto*c*. 2015;10(12):1904–1914. 10.1038/nprot.2015.125. 26513669

[72] Schindelin J, Arganda-Carreras I, Frise E, et al. Fiji: an open-source platform for biological-image analysis. Nat Method*s*. 2012;9(7):676–682. 10.1038/nmeth.2019. 22743772 PMC3855844

[73] Bouxsein ML, Boyd SK, Christiansen BA, Guldberg RE, Jepsen KJ, Müller R. Guidelines for assessment of bone microstructure in rodents using micro-computed tomography. J Bone Miner Re*s*. 2010;25(7):1468–1486. 10.1002/jbmr.141. 20533309

[74] Declercq H, Van den Vreken N, De Maeyer E, et al. Isolation, proliferation and differentiation of osteoblastic cells to study cell/biomaterial interactions: comparison of different isolation techniques and source. Biomaterial*s*. 2004;25(5):757–768. 10.1016/S0142-9612(03)00580-5. 14609664

