# Supplementary material for: Sphingosine-1-phosphate promotes osteogenesis by stimulating osteoblast growth and neovascularization in a vascular endothelial growth factor–dependent manner
Source: J Bone Miner Res. 2024 Jan 24;39(3):357–72. doi: 10.1093/jbmr/zjae006 (PMC11240155; doi:10.1093/jbmr/zjae006)
Supplement: wille_et_al_supplementary_information_revison3_zjae006 [file wille_et_al_supplementary_information_revison3_zjae006.pdf]

Supplementary Information

Sphingosine-1-Phosphate (S1P) promotes osteogenesis by stimulating osteoblast growth and neovascularization in a vascular endothelial growth factor (VEGF)-dependent manner

Annalena Wille[1], Sarah Weske[1], Karin von Wnuck Lipinski[1], Philipp Wollnitzke[1], Nathalie H. Schröder[1], Nadine Thomas[1], Melissa K. Nowak[1], Jennifer Deister-Jonas[1], Björn Behr[2], Petra Keul[1], *Bodo Levkau[1]

Supplementary Material and Methods:

Co-culture experiments

For co-culture experiments, BMEC were seeded in gelatine-coated cell-culture dished and cultivated in M1168 Complete Endothelial Cell Medium (Cell Biologics, Chicago, USA) for 24 hours. Afterward, the medium was exchanged with pOB differentiation medium supplemented with Endothelial Cell Growth Supplements (Cell Biologics, Chicago, USA) and pOBs were added at a 2:5 ratio to BMECs. S1P was added daily, and images were obtained using the CKX54 Microscope (Olympus, Shinjuku, Japan) after 24 h, 48 h and 72 h of co-cultivation.

For fluorescence staining of co-cultured cells, the cells were washed twice in PBS and fixed with 4 % PFA for 20 minutes. After an additional three washes with PBS buffer, fluorescently tagged antibodies against CD31 (AF-488, Biolegend, San Diego, USA, 1:100) and OSX (AF-548, Santa Cruz Biotechnology, Dellas, Texas, 1:100) were added in a staining solution containing 0.5 % Triton X, 0.1 mM CaCl2 and 2 % BSA in PBS for two hours. The cells were washed three times with PBS buffer and incubated for 5 minutes with 0.5 µg/mL DAPI solution (Thermo Fisher Scientific, Waltham, USA). After 3 washes in PBS for 5 minutes, sections are mounted using FlouromountG (Thermo Fisher Sciencific, Waltman, USA). Images were acquired using a Zeiss 880 LSM confocal microscope (Carl Zeiss, Jena, Germany).

Quantitative Real-Time PCR

Gene expression analysis was performed as described in the material and methods section. Additionally used primers are listed in Supplementary table 1.

Supplementary Table 1: Primers used for Quantitative Real-Time PCR

**Gene**	**Product/5‘-3‘Sequence**	**Company**
Bglap	Fw: GCGCTCTGTCTCTCTGACCT<br>Rv: ACCTTATTGCCCTCCTGCTT	Eurofins Genomics, Ebersberg, Germany
Sparc	Fw: GTGGAAATGGGAGAATTTGAGGA<br>Rv: CTCACACACCTTGCCATGTTT	Eurofins Genomics, Ebersberg, Germany
Postn	Fw: CCTGCCCTTATATGCTCTGCT<br>Rv: AAACATGGTCAATAGGCATCACT	Eurofins Genomics, Ebersberg, Germany
Sp7	Fw: ATGGCGTCCTCTCTGCTTG<br>Rv: TGAAAGGTCAGCGTATGGCTT	Eurofins Genomics, Ebersberg, Germany
Spp1	Fw: AGCAAGAAACTCTTCCAAGCAA<br>Rv: GTGAGATTCGTCAGATTCATCCG	Eurofins Genomics, Ebersberg, Germany

Bone Marrow Preparations of S1P measurements

To obtain bone marrow for S1P analysis, we retrieved one femur and removed adjacent tissue. We removed the epiphyseal regions to access the bone, which was then placed into a 0.5 mL centrifuge tube. An 18 G needle was used to make a hole at the bottom of the tube before putting it in a 1.5 mL microcentrifuge tube. After spinning the tubes at 10,000 g for 15 seconds. The bone marrow was weighted for normalization.

Finally, the bone marrow was resuspended in 200 µL MeOH. 10 µL of internal standard S1P was added. After overnight precipitation at -80 °C, the solution was centrifuged at 21,000 g for 10 minutes, and supernatants were collected for S1P quantification. The analysis was carried out following the prescribed procedure for plasma samples.

Supplementary Figures:

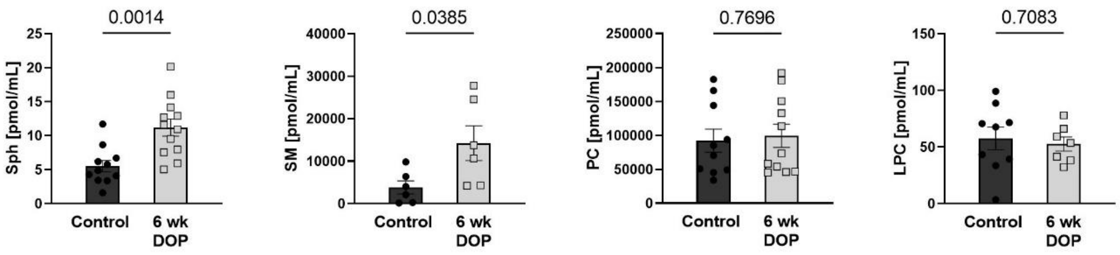

Supplementary Figure 1: Pharmacological inhibition of the S1P-Lyase leads to the accumulation of sphingolipids in plasma. Plasma levels of sphingosine (Sph) (n=11/12), sphingomyelin (SM) (n=6/6), phosphatidylcholine (PC) (n=10/11) and lysophosphatidylcholine (LPC) (n=9/7) of C57Bl6 control animals and after six weeks of DOP treatment (3 mg/lg/d) measured with LC-MS/MS. Data are presented as mean ± SEM, two-tailed t-test was used for statistical analysis.

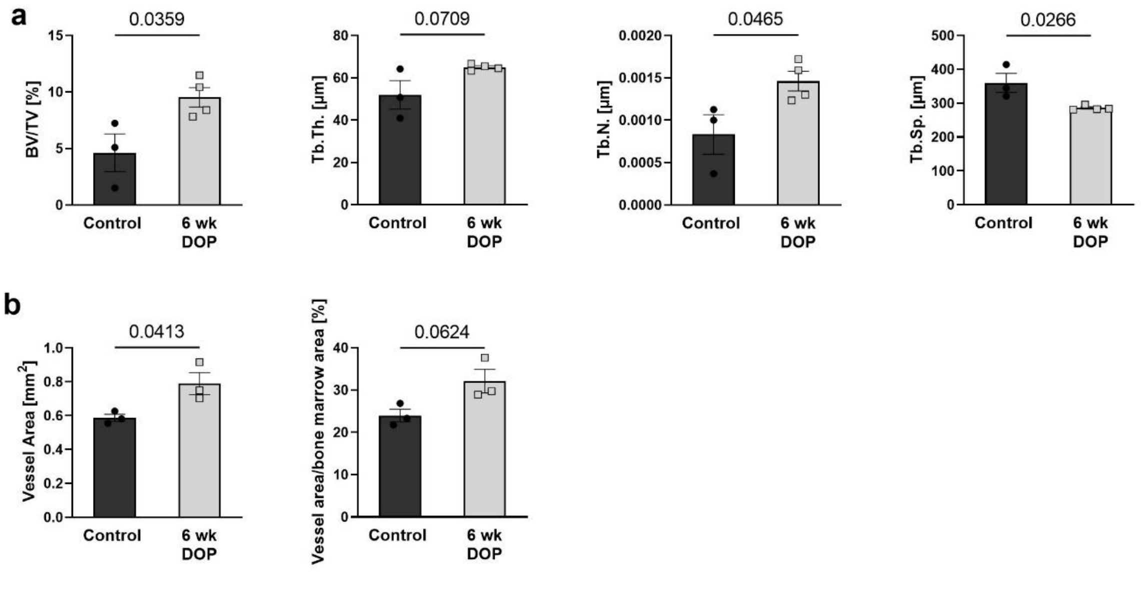

Supplementary Figure 2: DOP treatment increases bone volume and vessel density in female mice. (a) BV/TV, Tb.Th., Tb.N. and Tb.Sp of female C57BL/6J mice with and without 6 weeks of DOP treatment (3 mg/kg/d) (n=3/4) and (b) vessel area and vessel area per bone marrow area

assessed by endomucin staining if these animals (n=3/3). Data are presented as mean ± SEM, two-tailed t-test was used for statistical analysis.

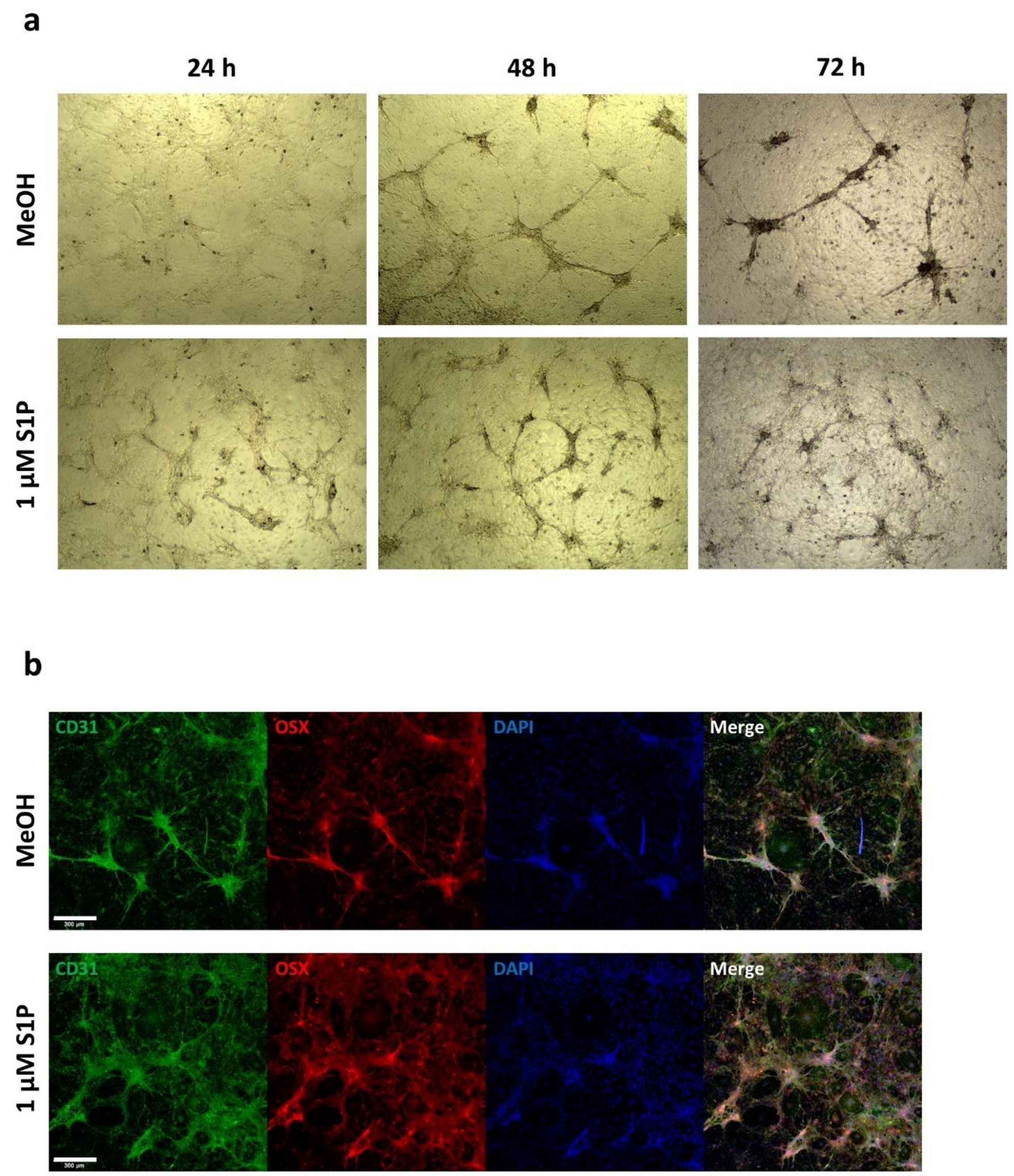

Supplementary Figure 3: pOB and BMEC co-culture enhances the formation of vessel-like structures. (a) Brightfield images of pOB and BMEC co-cultures after 24, 48 and 72 hours of cultivation and (b) fluorescence staining of CD31, Osterix (OSX) and DAPI after 72 hours of coculture with and without daily S1P treatment; scale bar = 300 µm.

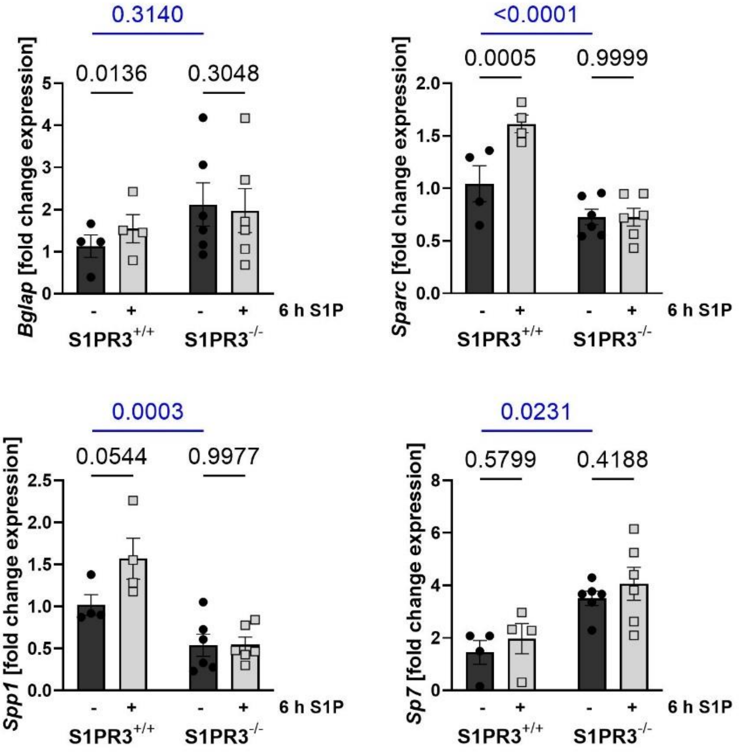

Supplementary Figure 4: S1P treatment leads to changes in gene expression of several osteogenic markers. Fold change gene expression levels of Osteocalcin (*Bglap*), Osteonectin (*Sparc*), Periostin (*Postn*), Osteopontin (*Spp1*) and Osterix (*Sp7*) in S1PR3$^{+/+}$ and S1PR3$^{-/-}$ osteoblasts with and without S1P treatment of 6 hours. Data are presented as mean ± SEM, a paired two-was ANOVA (black) or unpaired two-way ANOVA (blue) was used for statistical analysis.

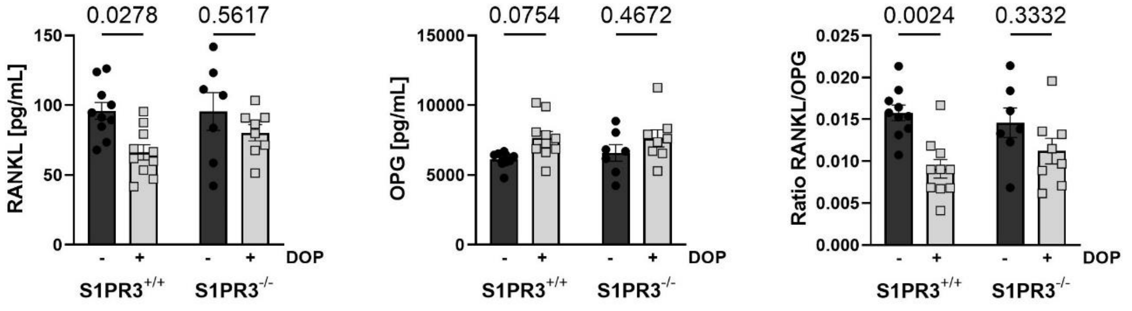

Supplementary Figure 5: The DOP mediated decrease in RANKL and increase in OPG is S1PR3 mediated. RANKL and OPG plasma levels and the RANKL/OPG ratio of S1PR3$^{+/+}$ control mice and after 6 weeks of DOP treatment (3 mg/kg/d) and in S1PR3$^{-/-}$ mice with the

same treatment (n=10/10/7/8). Data are presented as mean ± SEM, and a Two-way ANOVA was used for statistical analysis.

Supplementary Tables:

Supplementary Table 2: Interaction p-value, Column and Row factors determined by Two-way ANOVA corresponding to figure 3.

**Fig. 3e**	**p-Value**
Interaction	0.0843
S1P Treatment	0.0817
VEGFa-AB Treatment	0.1086
**Fig. 3i *Pdgfa***	**p-Value**
Interaction	0.0809
S1P Treatment	0.129
VEGFa-AB Treatment	0.0530
**Fig. 3i *Pdgfb***	**p-Value**
Interaction	0.1081
S1P Treatment	0.0063
VEGFa-AB Treatment	0.1545
**Fig. 3i *Tgfb***	**p-Value**
Interaction	0.0633
S1P Treatment	0.2020
VEGFa-AB Treatment	0.7216

Supplementary Table 3: Interaction p-value, Column and Row factors determined by Two-way ANOVA corresponding to figure 4.

**Fig. 4a**	**p-Value**
Interaction	0.0486
DOP Treatment	<0.0001
Axitinib Treatment	<0.0001
**Fig. 4c BV/TV**	**p-Value**
Interaction	0.6406
DOP Treatment	0.0006
Axitinib Treatment	0.3469
**Fig. 4c Tb.Th.**	**p-Value**
Interaction	0.6127
DOP Treatment	0.1243
Axitinib Treatment	0.3165
**Fig. 4c Tb.N.**	**p-Value**
Interaction	0.6378
DOP Treatment	0.0007
Axitinib Treatment	0.4343
**Fig. 4c Tb.Sp.**	**p-Value**
Interaction	0.2202
DOP Treatment	0.0002
Axitinib Treatment	0.1455
**Fig. 4e Ct.Th.**	**p-Value**
Interaction	0.0058
DOP Treatment	<0.0001
Axitinib Treatment	0.7130
**Fig. 4e Ct.Ar./Tt.Ar.**	**p-Value**
Interaction	<0.0001
DOP Treatment	<0.0001
Axitinib Treatment	0.9810
**Fig. 4h Stiffness**	**p-Value**
Interaction	0.2842
DOP Treatment	0.0011
Axitinib Treatment	0.0007
**Fig. 4h Ultimate Force**	**p-Value**
Interaction	0.0054
DOP Treatment	<0.0001
Axitinib Treatment	0.4631

Supplementary Table 4: Interaction p-value, Column and Row factors determined by Two-way ANOVA corresponding to figure 5.

**Fig. 5a** ***Vegfa***	**p-Value**
Interaction	0.0387
S1P Treatment	0.0034
TY-52156 Treatment	0.3889
**Fig. 5a VEGFa**	**p-Value**
Interaction	0.0374
S1P Treatment	0.0046
TY-52156 Treatment	0.0718
**Fig. 5b** ***Vegfa***	**p-Value**
Interaction	0.7660
S1P Treatment	0.0050
S1PR3 KO	0.5162
**Fig. 5b VEGFa**	**p-Value**
Interaction	0.1449
S1P Treatment	0.0564
S1PR3 KO	0.0004
**Fig. 5c**	p-Value
Interaction	0.1449
S1P Treatment	0.0564
S1PR3 KO	0.0004

Supplementary Table 5: Interaction p-value, Column and Row factors determined by Two-way ANOVA corresponding to figure 6.

**Fig. 6a**	**p-Value**
Interaction	0.1499
DOP Treatment	0.0034
S1PR3 KO	0.970
**Fig. 6b**	**p-Value**
Interaction	0.0036
DOP Treatment	0.2915
S1PR3 KO	0.0002
**Fig. 6d BV/TV**	**p-Value**
Interaction	0.2342
DOP Treatment	<0.0001
S1PR3 KO	<0.0001
**Fig. 6d Tb.Th.**	**p-Value**
Interaction	0.1929
DOP Treatment	0.0174
S1PR3 KO	<0.0001
**Fig. 6d Tb.N.**	**p-Value**
Interaction	0.4399
DOP Treatment	0.0006
S1PR3 KO	0.0007
**Fig. 6d Tb.Sp.**	**p-Value**
Interaction	0.7059
DOP Treatment	<0.0001
S1PR3 KO	0.8214
